# Hemorrhagic Pseudocyst of Pancreas Treated with Coil Embolization of Gastroduodenal Artery: A Case Report and Review of Literature

**DOI:** 10.1155/2015/480605

**Published:** 2015-12-27

**Authors:** Sudhir Kumar Jain, Vishnuraja Rajendran, Maneesh K. Jain, Ronal Kori

**Affiliations:** Department of Surgery, Maulana Azad Medical College, New Delhi 110002, India

## Abstract

Hemorrhage into pseudocyst of pancreas can rarely present as life threatening massive UGI bleeding. We present a case of 21-year-old male, admitted to our department, who was a known case of posttraumatic acute pancreatitis and who developed massive upper GI bleeding. CT angiography of abdomen showed aneurysm of gastroduodenal artery. Patient was successfully treated with coil embolization of gastroduodenal artery.

## 1. Introduction

Hemorrhage into the pseudocyst is one of the rare and life threatening complications of pseudocyst of pancreas. Hemorrhage is due to the erosion of the arterial walls abutting the pseudocyst. Splenic artery is most commonly involved. Occasionally, the pseudocyst of the pancreas may communicate with the adjacent bowel, that is, duodenum, and such type of bleeding can present rarely as massive upper gastrointestinal bleeding.

We present a case of hemorrhagic pseudocyst of pancreas presenting as massive upper GI bleeding, managed successfully with selective percutaneous intra-arterial coil embolization along with relevant review of the literature.

## 2. Case Presentation

A 21-year-old male who was previously treated in our hospital for posttraumatic acute pancreatitis 2 months ago came to the emergency department with complaints of abdominal pain associated with nonpassage of flatus and feces for 4 days. He gave history of passing bright red blood per rectum for 2 days. On examination, patient general condition was fair with normal vital signs. Abdomen was mildly distended with generalized tenderness present over the abdomen without any guarding or rigidity. Free fluid was present in abdomen and bowel sounds were sluggish. The rest of the systemic examination was within normal limits.

Laboratory examinations showed normal blood counts, hemoglobin was 11 gm%, and the liver function tests were within normal limits. The ascitic fluid tapping was done and sent for biochemical and histological examination. It showed amylase of 6366 IU/cumm, glucose of 63 gms%, and protein of 3.8 gms%. Histological examination of ascitic fluid showed a TLC of 2160/cumm, among which polymorphs form 66% and lymphocytes are 34%.

Initially the patient was managed conservatively but during the 6th day of admission patient developed an episode of massive episode of hematemesis and hemoglobin level fell to Hb-4 gm%. Patient was resuscitated with 4 units of whole blood transfusion. Hemoglobin was built up to 8.4 gm%. Patient underwent UGI endoscopy which showed a large extrinsic bulge seen in antrum along the lesser curvature with white based ulcers approximately 2 × 0.5 cm with erythematous margin seen over bulge near pylorus without any active ooze/bleeding. Patient had another episode of massive hematemesis two days later which lowered the hemoglobin to 4.6 gm%. Patient was resuscitated with blood and IVF, and hemoglobin was built up to 9.1 gm%.

**Figure 1:**
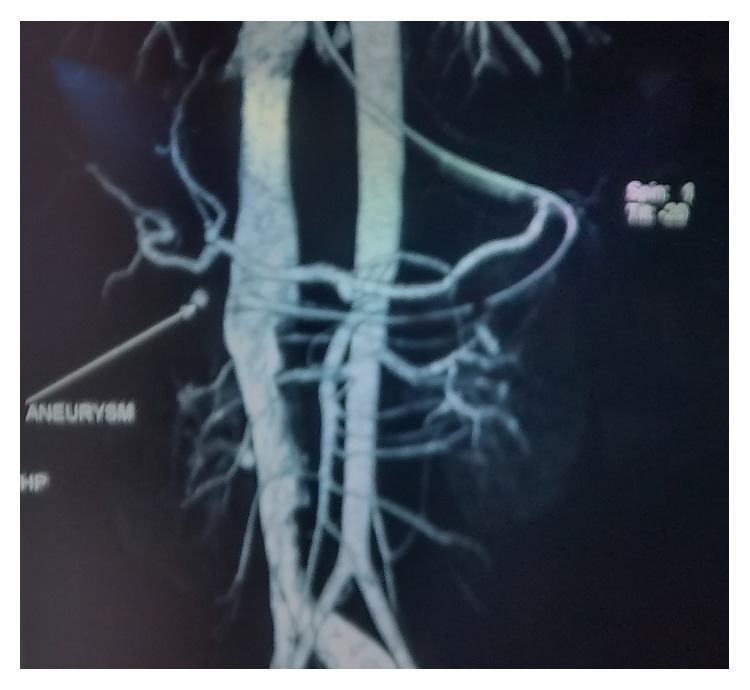
CT abdominal angiography showing the aneurysm of gastroduodenal artery.

**Figure 2:**
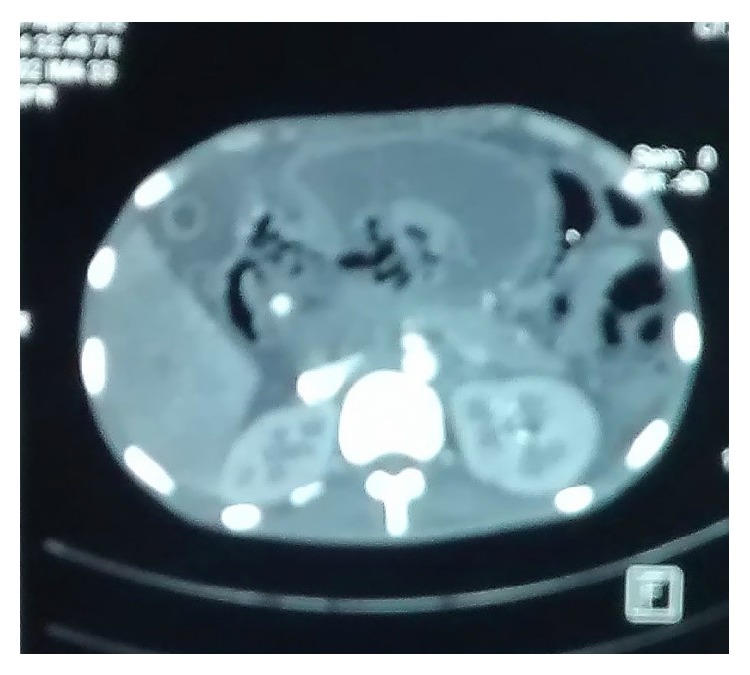
CT abdominal angiography showing gastroduodenal artery aneurysm abutting the wall of the pseudocyst of pancreas.

CT abdominal angiography was done to identify the source of bleeding which showed a large collection replacing neck of pancreas (likely pseudocyst) in the gastrohepatic region with heterogeneous contents (likely hemorrhage/debris) with multiple air foci within, which is communicating with the lumen of antropyloric region/proximal duodenum. It also showed a pseudo aneurysm of gastroduodenal artery abutting the wall of the pseudocyst with extravasation of contrast (Figures 1 and 2). Patient was taken for gastroduodenal embolization. Using transfemoral approach, under local anaesthesia, 5 Fr introducer was inserted up to the celiac trunk. Selective cannulation of celiac trunk and arteriogram was done. Pseudoaneurysm of gastroduodenal artery was noted. Selective cannulation of common hepatic artery and gastroduodenal artery was done. 0.035-inch stainless steel metallic macrocoils were used to embolize the gastroduodenal artery. Procedure was uneventful. After procedure, patient had no further episodes of hematemesis and symptomatically improved before discharge on proton pump inhibitors. The repeat endoscopy after 3 months of discharge revealed healed ulcer.

## 3. Discussion

According to the Atlanta Classification 2012, pseudocyst of the pancreas develops 4 weeks after the development of acute pancreatitis. Infection, bleeding, obstruction, and rupture are some of the complications of pseudocyst of pancreas [1].

Hemorrhage into the pseudocyst occurs due to the erosion of the arterial walls abutting the pseudocyst due to the enzymatic action of the fluid present in the pseudocyst. Splenic artery is the most commonly involved (30–50%), followed by the gastroduodenal (17%) and pancreaticoduodenal arteries (11%) [2].

Fistulous communication may occur between the pseudocyst and the nearby viscus. Hemorrhage into such a pseudocyst may present as a rare cause of UGI bleeding with reported incidence of 1.7–2.5% [3]. When the haemorrhagic pseudocyst ruptures into the peritoneum, biliary tract, or retroperitoneum, it can present as hemosuccus pancreatitis, hemobilia, and retroperitoneal bleeding, respectively [4].

Hemorrhagic pseudocyst of pancreas carries a very high mortality of 40%. Arterial embolization is used as the temporary or the definite procedure for the treatment of haemorrhagic pseudocyst. Since arterial embolization has chances of rebleeding, some advocate embolization as only a temporary procedure before definitive surgery. Carr et al. (2000) advocate percutaneous arterial embolization for hemodynamically stable patients and surgery for patients who are actively bleeding, hemodynamically unstable patients, and patients with failed embolization or with other complications like infection or extrinsic compression [5].

Conservative medical management in cases of hemorrhagic pseudocyst of pancreas has always been nearly fatal [6].

Various surgical procedures have been tried in the management of bleeding pseudoaneurysm of pancreas with limited success. Pseudoaneurysm resection followed by arteriorrhaphy, partial pancreatectomy, splenectomy, ligation of bleeder, and pseudocyst drainage are some surgical procedures tried for controlling bleeding pseudocyst of pancreas. Morbidities associated with surgical procedures are high compared to percutaneous arterial embolization [2, 6–9].

With the current technologies, timely identification of the source of bleeding with CT angiography, and immediate selective percutaneous arterial embolization, more lives can be saved than ever before for a disease with no medical therapy and unacceptable mortality.
